# Reproducibility of quantitative coronary computed tomography angiography in asymptomatic individuals and patients with acute chest pain

**DOI:** 10.1371/journal.pone.0207980

**Published:** 2018-12-14

**Authors:** Martina C. de Knegt, Morten Haugen, Jesper J. Linde, Jørgen Tobias Kühl, Børge G. Nordestgaard, Lars V. Køber, Jens D. Hove, Klaus F. Kofoed

**Affiliations:** 1 Department of Cardiology, Rigshospitalet, Faculty of Health Sciences, University of Copenhagen, Copenhagen, Denmark; 2 Department of Cardiology, Hvidovre Hospital, Faculty of Health Sciences, University of Copenhagen, Copenhagen, Denmark; 3 Department of Clinical Biochemistry, Herlev Hospital, Faculty of Health Sciences, University of Copenhagen, Copenhagen, Denmark; 4 Department of Radiology, Rigshospitalet, Faculty of Health Sciences, University of Copenhagen, Copenhagen, Denmark; Kurume University School of Medicine, JAPAN

## Abstract

**Purpose:**

Quantitative computed tomography (QCT) provides important prognostic information of coronary atherosclerosis. We investigated intraobserver and interobserver QCT reproducibility in asymptomatic individuals, patients with acute chest pain without acute coronary syndrome (ACS), and patients with acute chest pain and ACS.

**Methods:**

Fifty patients from each cohort, scanned between 01/02/2010-14/11/2013 and matched according to age and gender, were retrospectively assessed for inclusion. Patients with no coronary artery disease, previous coronary artery bypass graft surgery, and poor image quality were excluded. Coronary atherosclerosis was measured semi-automatically by 2 readers. Reproducibility of minimal lumen area (MLA), minimal lumen diameter (MLD), area stenosis, diameter stenosis, vessel remodeling, plaque eccentricity, plaque burden, and plaque volumes was assessed using concordance correlation coefficient (CCC), Bland-Altman, coefficient of variation, and Cohen’s kappa.

**Results:**

A total of 84 patients (63 matched) were included. Intraobserver and interobserver reproducibility estimates were acceptable for MLA (CCC = 0.94 and CCC = 0.91, respectively), MLD (CCC = 0.92 and CCC = 0.86, respectively), plaque burden (CCC = 0.86 and CCC = 0.80, respectively), and plaque volume (CCC = 0.97 and CCC = 0.95, respectively). QCT detected area and diameter stenosis ≥50%, positive remodeling, and eccentric plaque with moderate-good intraobserver and interobserver reproducibility (kappa: 0.64–0.66, 0.69–0.76, 0.46–0.48, and 0.41–0.62, respectively). Reproducibility of plaque composition decreased with decreasing plaque density (intraobserver and interobserver CCC for dense calcium (>0.99; 0.98), fibrotic (0.96; 0.93), fibro-fatty (0.95; 0.91), and necrotic core tissue (0.89; 0.84). Reproducibility generally decreased with worsening clinical risk profile.

**Conclusions:**

Semi-automated QCT of coronary plaque morphology is reproducible, albeit with some decline in reproducibility with worsening patient risk profile.

## Introduction

Multidetector computed tomography (MDCT) is a guideline recommended non-invasive imaging modality for the assessment of obstructive coronary artery disease (CAD)[1]. Recent advancements in software technology now allow for semi-automated quantitative assessment of coronary atherosclerosis, thereby providing a detailed description of coronary plaque morphology with the potential for improved reproducibility and accuracy compared to traditional qualitative MDCT assessments[2].

Good reproducibility of quantitative computed tomography (QCT) is important for diagnostic purposes and for risk-stratification purposes in various patient populations[3–5]. Coronary plaque morphology has, however, been shown to differ in different patient populations, with the majority of research conducted in patients with stable angina and in patients with acute coronary syndrome (ACS)[6,7]. It can be hypothesized that these differences may affect QCT reproducibility assessments as correct identification of mild levels of coronary disease, especially non-calcified plaque[2], as well as correct discernment of lumen and vessel contours in severe levels of coronary disease, especially combined with even slight motion, noise, or blooming artifacts, can be difficult. Studies investigating intraobserver and interobserver reproducibility using semi-automated QCT are, however, few and reproducibility has only been investigated in relatively small numbers of highly selected patients with low-intermediate plaque burdens[8–11].

In this study, we investigated intraobserver and interobserver QCT reproducibility in asymptomatic individuals, patients with acute chest pain without ACS, and patients with acute chest pain and ACS.

## Material and methods

### Study population

All participants included in this study underwent MDCT at The Department of Radiology, Rigshospitalet, Copenhagen, Denmark. All patients gave written informed consent to have a MDCT performed for research purposes and the local ethics committee approved individual study protocols. This study was approved by *The Danish Data Protection Agency* and all procedures performed in studies involving human participants were in accordance with the ethical standards of the institutional and/or national research committee and with the 1964 Helsinki declaration and its later amendments or comparable ethical standards.

Asymptomatic individuals from the general population were recruited from *The Copenhagen General Population Study*, *CGPS*[12]. Patients presenting with acute chest pain but without signs of ACS were recruited from *Cardiac CT in the Treatment of Acute Chest Pain*, *CATCH* (clinical trial number NCT01534000)[13]. Patients fulfilling criteria for either unstable angina pectoris or non-ST elevation myocardial infarction were recruited from *Very Early versus Deferred Invasive Evaluation using Computerized Tomography in Patients with Acute Coronary Syndromes*, *VERDICT* (clinical trial number NCT02061891).

Fifty patients were randomly selected from the *VERDICT* cohort and subsequently matched according to age and gender with participants from the *CGPS* and *CATCH* trial, as described in a previous study where all 150 participants have been reported[2]. This prior study dealt with the reproducibility of qualitatively assessed coronary atherosclerosis[2]. Matching was prioritised in this study as increasing burden of CAD and increasing calcification with age and gender are well known factors that could influence reproducibility estimates if not accounted for. Of these 150 participants scanned between 01/02/2010-14/11/2013, participants without coronary artery disease (as assessed by 2 readers) and with coronary artery bypass grafts (CABG) did not undergo QCT. Furthermore, MDCT datasets with image quality deemed unsuitable for semi-automated plaque quantification due to severe motion, noise, calcification, pacemaker artifacts, field of view problems, and poor contrast timing, were excluded as previously described[14].

MDCT image data, medical history and cardiovascular risk profile were acquired from the respective study databases. In this study, participants were divided into three subpopulations based on clinical presentation: *Asymptomatic* (*CGPS* participants), *Acute chest pain–(minus) ACS* (*CATCH* participants), and *Acute chest pain + ACS* (*VERDICT* participants).

### MDCT scan protocol

All participants with a heart rate >60 beats per minute (bpm) and no contraindications to beta blockade were pretreated with oral beta blockers according to a standardised protocol. If necessary, intravenous beta blocker (*CATCH* and *VERDICT* participants) was given just before scanning. Patients received 0.8 mg nitroglycerin prior to scanning if no contraindications were present. All participants were in sinus rhythm during scanning.

Image acquisition was performed using a 320-slice MDCT (Aquilion one, Vision Edition, Canon, Tokyo, Japan). Overall scan protocol: 320 x 0.5 mm detector collimation, a median (interquartile range (IQR)) tube voltage of 120 (100, 120) kV (body mass index (BMI) dependent), a median (IQR) tube current of 450 (410, 500) mA, and a gantry rotation time of median (IQR) of 0.35 (0.35, 0.35) s (heart rate dependent). Intravenous contrast media (Visipaque, GE Healthcare, Chalfont St. Giles, United Kingdom (*CGPS* and *VERDICT*) or Omnipaque, GE Healthcare, Chalfont St. Giles, United Kingdom (*CATCH*)) was infused with a flow rate of 5.0–6.0 ml/s (weight dependent) with a biphasic injection protocol followed by a saline chaser. The automatic bolus triggering technique was used for initiating image acquisition. Reconstructions at best phase of the R-R interval using an automatic raw data motion analysis tool (PhaseXact, Toshiba) were performed. Images were reconstructed with 0.5 mm slice thickness and increments of 0.25 mm. The conversion factor 0.014 mSv/(mGyxcm) was used to calculate the effective radiation dose.

### Quantitative plaque analysis

QCT of coronary atherosclerotic plaque per segment was performed in a semi-automated manner using dedicated software (QAngio CT Research Edition version 2.02, Medis Medical Imaging Systems, Leiden, The Netherlands) by 2 readers with 1 year and 3 years of reading experience, respectively. Coronary tree extraction with the generation of multiplanar reformatted (MPR) volumes using an automatic tree extraction algorithm[15] and coronary vessel segmentation (in accordance with *Society of Cardiovascular Computed Tomography (SCCT)* guidelines[1]) was performed by the primary reader. If no vessel branches were present to indicate a given segment border, vessel lengths were divided up into thirds to provide proximal, mid, and distal segments. The extracted coronary tree with segment borders (but no vessel wall or lumen contours) was then loaded and assessed by reader 1 twice with a 3 month interval (measurement 1 and measurement 2) for the assessment of intraobserver reproducibility and by reader 2 (blinded) for the assessment of interobserver reproducibility. Calculation of interobserver reproducibility was done using measurement 2 by reader 1 and the measurements by reader 2.

**Fig 1:**
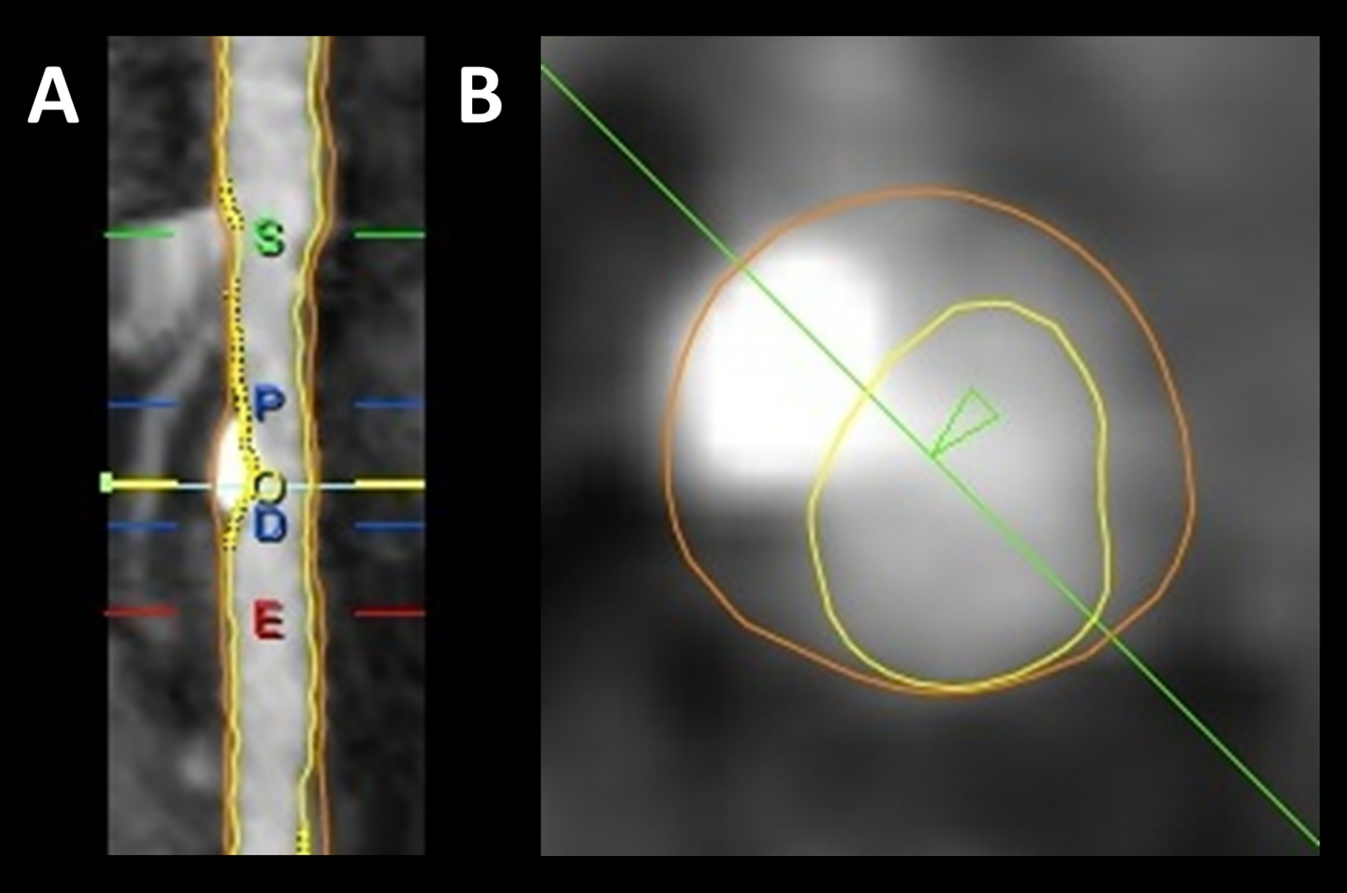
Example of quantitative analysis of a proximal left anterior descending artery segment. A. Longitudinal straightened multiplanar reconstruction where *S* and *E* are the start and end of the segment, respectively; *P* and *D* are the proximal and distal borders of the lesion, respectively; *O* is the point of maximal obstruction. B. Transverse vessel view at the point of maximal obstruction.

Each reader performed automatic lumen and outer vessel wall contour registration[16,17] followed by manual editing in longitudinal and transverse vessel views of both the lumen and outer vessel wall contours when needed. As determined by each reader, coronary segments of included individuals with motion, severe image noise, severe calcification, <1.5 mm in vessel diameter, occlusions with limited retrograde contrast filling, and segments with stents traversing the entire length of a given segment were not assessed. Furthermore, only segments with coronary atherosclerotic plaque (of any degree) underwent QCT assessment: If a reader determined that there was coronary plaque in a given segment, the reader determined the proximal and distal borders of the lesion visually and QCT parameters were automatically calculated for the lesion, see Fig 1. An automatic reference method where a regression analysis is used on the whole lesion to obtain a linear reference was used in the calculation of remodeling index (RI), area stenosis and diameter stenosis.

Plaque composition was assessed using Hounsfield unit (HU) cut-off values adapted to lumen attenuation[18]. This is based on the principle that lumen intensities influence plaque intensity and decrease along the length of a vessel, are lower in vessel segments with severe stenoses, and are higher in vessel segments with calcified plaque due to blooming artifacts[17–19]. Definitions of QCT derived parameters are given in Table 1.

**Table 1:** Definitions of quantitative computed tomography (QCT) derived parameters.

QCT parameter			Definition
**Lumen and vessel geometry**		Minimal lumen area (MLA), mm^2^	The minimal lumen area at the point of maximal obstruction
		Area stenosis, %	= 1-(MLA/reference lumen area) x 100
		Minimal lumen diameter (MLD), mm	The minimal lumen diameter at the point of maximal obstruction
		Diameter stenosis, %	= 1-(MLD/reference lumen diameter) x 100
		Remodeling index	= vessel area at the point of maximal obstruction/reference vessel area
**Coronary plaque parameters**	**Distribution and burden**	Eccentricity index	At the point of maximal obstruction: = (maximum plaque thickness-minimum plaque thickness)/maximum plaque thickness
		Plaque burden, %	Between the proximal and distal ends of the coronary lesion: = (vessel volume-lumen volume)/vessel volume x 100
	**Volume and composition**	Plaque volume, mm^3^	Between the proximal and distal ends of the coronary lesion: = vessel volume-lumen volume
		Fibrotic volume, mm^3^	The volume based on all the pixel area measurements of the fibrous plaque category between the proximal and distal ends of the coronary lesion
		Fibro-fatty volume, mm^3^	The volume based on all the pixel area measurements of the fibro-fatty plaque category between the proximal and distal ends of the coronary lesion
		Necrotic core volume, mm^3^	The volume based on all the pixel area measurements of the necrotic core plaque category between the proximal and distal ends of the coronary lesion
		Dense calcium volume, mm^3^	The volume based on all the pixel area measurements of the dense calcium plaque category between the proximal and distal ends of the coronary lesion

Data are reported on both a lesion and patient basis (supplementary material) for the total population and for the subset of age and gender matched individuals.

### Statistics

Continuous normally distributed data are presented as mean (standard deviation, SD) and continuous non-normally distributed data as median (IQR). Categorical data are presented as absolute numbers (percentages). One-way analysis of variance and the Kruskall-Wallis test were used to assess differences in normally distributed data and non-normally distributed data, respectively. The χ2-test was used to test for differences in categorical data. Intraobserver and interobserver reproducibility estimates were analysed using Lin’s concordance correlation coefficient (CCC with 95% confidence intervals (CI))[20]. Furthermore, Bland Altman analyses, with mean bias (95% CI) and 95% limits of agreement, were conducted[21]. Normality of mean differences of all parameters was tested. Furthermore, the coefficient of variation (CV), a measure of variability relative to the mean, was calculated as (SD of the mean difference/total average of the mean values for each pair)x100. Intraobserver and interobserver reproducibility was calculated in all participants and across subpopulations. Reproducibility for categorical variables was assessed using Cohen’s Kappa (κ) and were interpreted as: absence of agreement ≤0; poor agreement 0.00–0.20; fair agreement 0.21–0.40; moderate agreement 0.41–0.60; good agreement 0.61–0.80; and excellent agreement >0.80[22]. All statistical analyses were performed using SPSS version 22, SPSS Inc., Chicago, IL, USA). Graphical illustrations were constructed using GraphPad Prism version 6.01 for Windows, GraphPad Software, San Diego California USA. *P*-values <0.05 were considered statistically significant.

## Results

### Patient population

**Fig 2:**
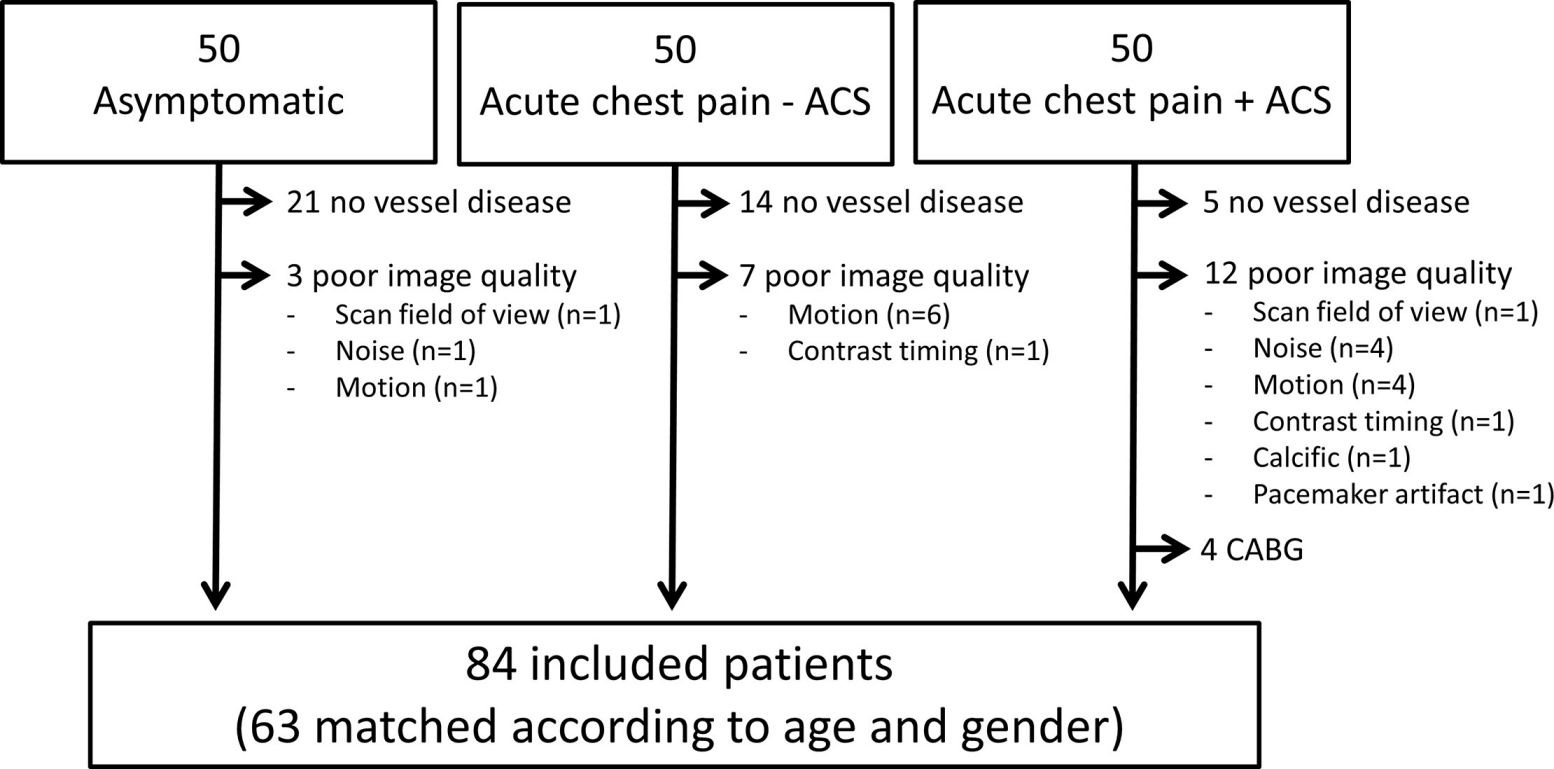
Flow chart of patient inclusion. ACS acute coronary syndrome; CABG coronary artery bypass graft.

Out of 150 selected participants, a total of 84 participants were included in this study, see Fig 2. Of these 84 included participants, 63 were matched 1:1 according to age and gender. Baseline characteristics for all 84 participants and for the three matched subpopulations are shown in Table 2. Of the 1428 segments available for analysis, segments with no atherosclerosis, motion, noise, severe calcification, vessel diameter <1.5 mm, limited retrograde contrast filling after occlusion, and stents were excluded and 343 and 335 paired measurements were obtained for intraobserver and interobserver assessment, respectively.

**Table 2:** Baseline characteristics.

	*All*	Asymptomatic	Acute chest pain—ACS	Acute chest pain + ACS	*p-value*
n	*84*	21	21	21	
Age, mean (SD)	*66*.*2 (8*.*4)*	66.7 (8.2)	66.2 (8.4)	65.8 (8.0)	*-*
Male, n (%)	*51 (61)*	14 (67)	14 (67)	14 (67)	*-*
Family history of CAD, n (%)	*24 (35)*	4 (27)	2 (10)	10 (63)	*<0*.*01*
Hypertension, n (%)	*48 (57)*	15 (71)	12 (57)	12 (57)	*0*.*55*
Hypercholesterolemia, n (%)	*39 (48)*	9 (43)	13 (62)	9 (47)	*0*.*44*
Diabetes, n (%)	*9 (11)*	1 (5)	3 (14)	4 (19)	*0*.*37*
Current smoking, n (%)	*25 (30)*	3 (14)	10 (48)	3 (16)	*<0*.*05*
Known CAD, n (%)	*16 (19)*	2 (10)	8 (38)	4 (19)	*0*.*08*
Prior AMI, n (%)	*14 (17)*	2 (10)	6 (29)	4 (19)	*0*.*29*
Height, mean (SD)	*173*.*2 (9*.*4)*	173.2 (9.0)	173.0 (9.3)	175.9 (10.1)	*0*.*58*
Weight, mean (SD)	*79*.*9 (16*.*9)*	78.0 (12.4)	83.6 (18.1)	81.9 (18.2)	*0*.*53*
BMI, mean (SD)	*26*.*4 (4*.*0)*	25.9 (3.3)	27.7 (4.6)	26.2 (3.7)	*0*.*29*
<50% stenosis, n (%)	*49 (58)*	18 (86)	13 (62)	6 (29)	*<0*.*01*
1 vessel disease, n (%)	*21 (25)*	3 (14)	4 (19)	9 (43)	
2 vessel disease, n (%)	*12 (14)*	0 (0)	3 (14)	6 (29)	
3 vessel disease, n (%)	*2 (2)*	0 (0)	1 (5)	0 (0)	
Heart rate, mean (SD)	*58*.*8 (7*.*3)*	58.7 (6.6)	60.8 (10.2)	59.3 (5.0)	*0*.*67*
Contrast (ml), median (IQR)	*80 (70*, *80)*	80 (80, 80)	70 (70, 80)	90 (70, 90)	*<0*.*05*
Effective radiation dose (mSv), median (IQR)	*2*.*8 (1*.*5*, *3*.*9)*	1.5 (1.3, 1.6)	3.6 (2.9, 6.1)	3.3 (2.8, 3.9)	*<0*.*001*

CAD coronary artery disease; AMI acute myocardial infarction; BMI body mass index; SD standard deviation; IQR interquartile range

### Geometrical vessel and lumen parameters

**Fig 3:**
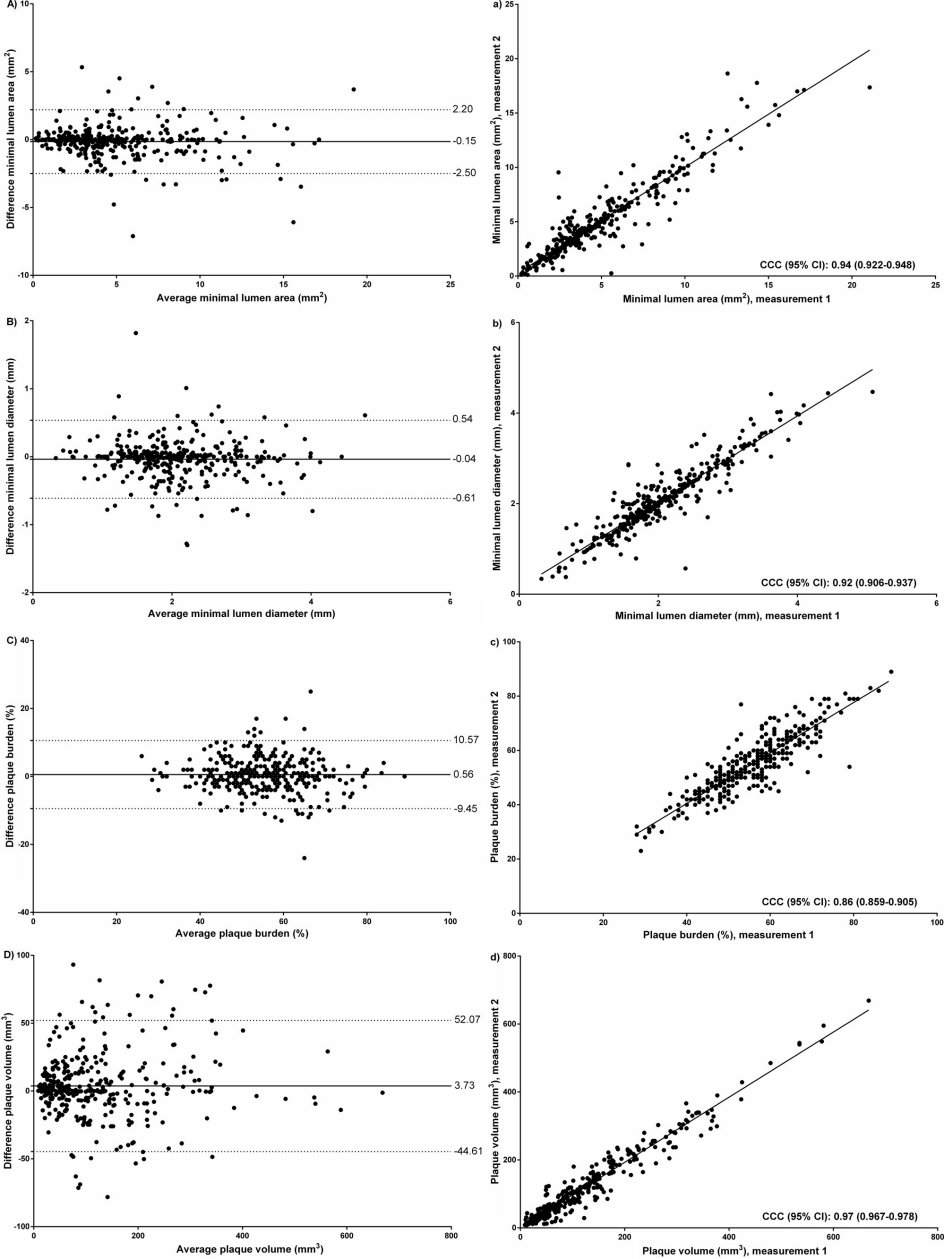
Intraobserver reproducibility (lesion basis) for minimal lumen area, minimal lumen diameter, plaque burden, and plaque volume. A-D show Bland Altman plots for minimal lumen area, minimal lumen diameter, plaque burden, and plaque volume, respectively; a-d show correlations for minimal lumen area, minimal lumen diameter, plaque burden, and plaque volume, respectively.

**Fig 4:**
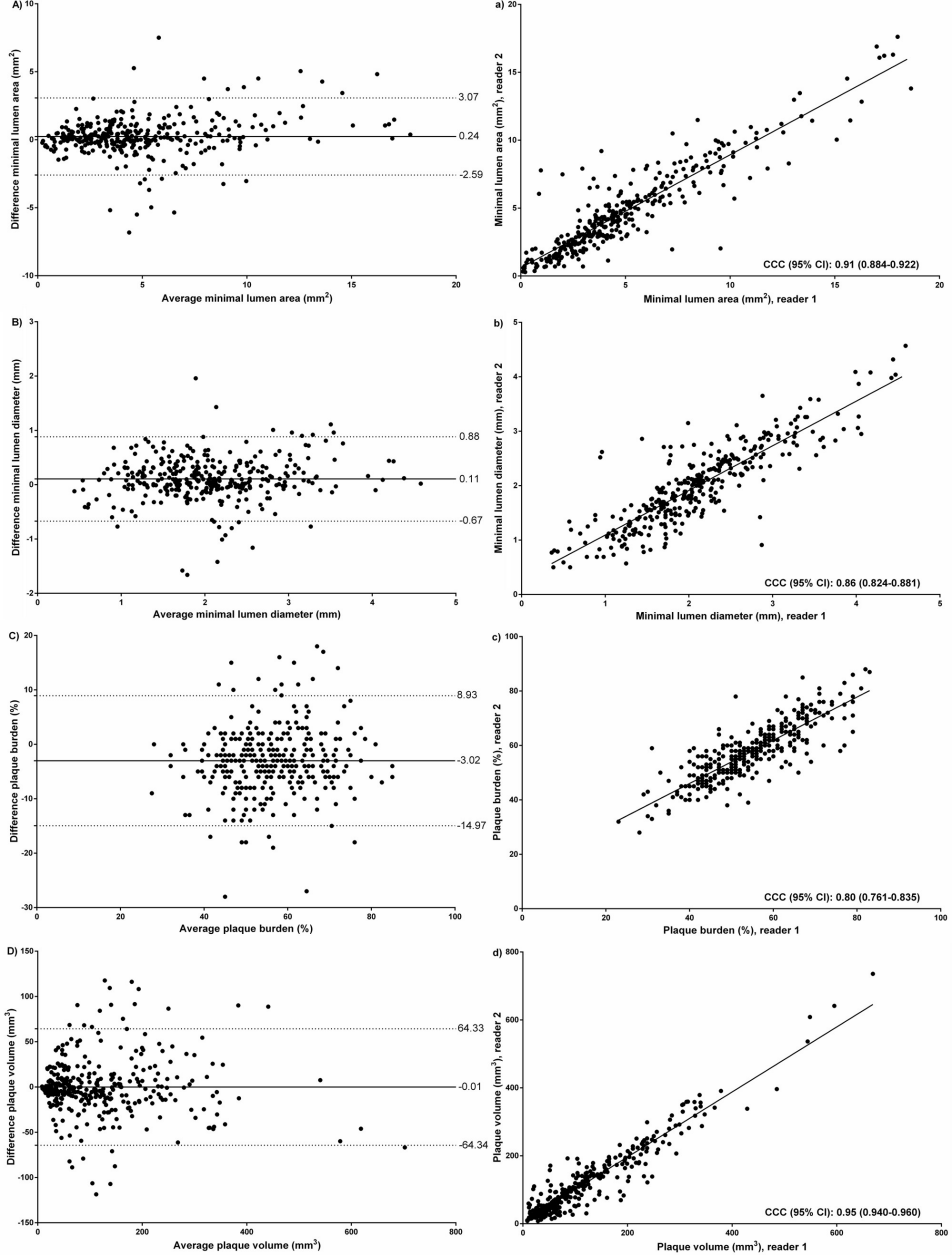
Interobserver reproducibility (lesion basis) for minimal lumen area, minimal lumen diameter, plaque burden, and plaque volume. A-D show Bland Altman plots for minimal lumen area, minimal lumen diameter, plaque burden, and plaque volume, respectively; a-d show correlations for minimal lumen area, minimal lumen diameter, plaque burden, and plaque volume, respectively.

Reproducibility estimates for minimal lumen area (MLA), area stenosis, minimal lumen diameter (MLD), and diameter stenosis are shown in Table 3, and Figs 3 and 4. MLA and MLD had good intraobserver and interobserver reproducibility with acceptable CCC and CV values, mean biases close to zero, and narrow limits of agreement. Reproducibility for area stenosis and diameter stenosis was weaker than their reference independent counterparts and limits of agreement for area stenosis and diameter stenosis ranged between -23.5 to 23.5% and -17.1 to 17.3% for intraobserver reproducibility and between -29.9 to 29.3% and -21.8 to 21.4% for interobserver reproducibility, respectively.

**Table 3:** Intraobserver and interobserver reproducibility estimates on a lesion basis.

	Intraobserver reproducibility n = 343 lesions					Interobserver reproducibility n = 335 lesions				
	CCC	95% CI	Mean bias±1.96SD	95% CI of mean bias	CV (%)	CCC	95% CI	Mean bias±1.96SD	95% CI of mean bias	CV (%)
Min. lumen area, mm^2^	0.94	0.922–0.948	-0.2±2.4	-0.3; 0.0	24	0.91	0.884–0.922	0.2±2.8	0.1; 0.4	29
Area stenosis, %	0.84	0.806–0.868	0.0±23.5	-1.3; 1.3	29	0.77	0.718–0.807	-0.3±29.6	-1.9; 1.3	36
Min. lumen diameter, mm	0.92	0.906–0.937	0.0±0.6	-0.1; 0.0	14	0.86	0.824–0.881	0.1±0.8	0.1; 0.2	19
Diameter stenosis, %	0.85	0.814–0.874	0.1±17.2	-1.0; 0.8	34	0.77	0.725–0.812	-0.2±21.6	-1.4; 1.0	43
Remodeling index	0.57	0.489–0.634	0.0±0.3	0.0; 0.0	17	0.53	0.447–0.602	0.0±0.3	0.0; 0.0	17
Eccentricity index	0.64	0.576–0.700	0.0±0.2	0.0; 0.0	15	0.58	0.504–0.646	0.0±0.3	0.0; 0.0	17
Plaque burden, %	0.86	0.859–0.905	0.6±10.0	0.0; 1.1	9	0.80	0.761–0.835	-3.0±12.0	-3.7; -2.4	11
Plaque volume, mm^3^	0.97	0.967–0.978	3.7±48.3	1.1; 6.4	20	0.95	0.940–0.960	0.0±64.3	-3.5; 3.5	27
Dense calcium, mm^3^	>0.99	0.995–0.997	0.2±6.5	-0.1; 0.6	13	0.98	0.973–0.981	-3.3±13.0	-4.0; -2.6	25
Fibrotic, mm^3^	0.96	0.953–0.969	2.8±28.8	1.3; 4.4	23	0.93	0.919–0.946	0.9±37.9	-1.2; 2.9	31
Fibro-fatty, mm^3^	0.95	0.935–0.957	0.6±13.9	-0.2; 1.3	30	0.91	0.887–0.924	1.5±17.6	0.6; 2.5	39
Necrotic core, mm^3^	0.89	0.868–0.912	0.1±8.5	-0.4; 0.6	50	0.84	0.804–0.866	0.8±9.0	0.3; 1.3	57

CCC concordance correlation coefficient; SD standard deviation; CV coefficient of variation; CI confidence interval

Using a binary approach, reproducibility of area and diameter stenosis (using a cut-off of 50% to identify lesions with and without significant stenosis) was found to be good for both intraobserver reproducibility (area stenosis κ (95% CI): 0.66 (0.55; 0.77), *p*<0.001; diameter stenosis κ (95% CI): 0.76 (0.65; 0.87), *p*<0.001) and interobserver reproducibility (area stenosis κ (95% CI): 0.64 (0.53; 0.74), *p*<0.001; diameter stenosis κ (95% CI): 0.69 (0.58; 0.80), *p*<0.001).

Reproducibility estimates of the RI on a continuous scale were weak (intraobserver and intraobserver CCC of 0.53 and 0.57, respectively), Table 3. Using a binary approach, reproducibility of positive remodeling (using the standard cut-off of ≥1.10 to identify positively remodelled lesions) was moderate for both intraobserver reproducibility (κ (95% CI): 0.48 (0.36; 0.60), *p*<0.001) and interobserver reproducibility (κ (95% CI): 0.46 (0.34; 0.58), *p*<0.001).

### Plaque eccentricity and plaque burden

Reproducibility estimates of the eccentricity index (EI) on a continuous scale were weak (intraobserver and interobserver CCC of 0.64 and 0.58, respectively), Table 3. Using a binary approach, reproducibility of eccentric plaque (defined as EI ≥0.67, corresponding to maximal plaque thickness thrice that of minimal plaque thickness at the site of maximal obstruction) was good for intraobserver reproducibility (κ (95% CI): 0.62 (0.52; 0.73), *p*<0.001) and moderate for interobserver reproducibility (κ (95% CI): 0.41 (0.30; 0.52), *p*<0.001).

Reproducibility estimates for plaque burden showed acceptable intraobserver and interobserver reproducibility with CCCs ranging from 0.80–0.86, mean biases close to zero, relatively narrow limits of agreement, and good CVs, Table 3, Figs 3 and 4.

### Plaque volume and plaque composition

Reproducibility estimates of plaque volumes on a lesion basis and patient basis for both intraobserver and interobserver reproducibility were good, Table 3, S1 Table, Figs 3 and 4. With regards to plaque composition for both intraobserver and interobserver reproducibility, decreasing CCCs and increasing CVs were seen with decreasing coronary plaque density.

### Reproducibility estimates across subpopulations

Reproducibility estimates of coronary plaque parameters across subpopulations on a lesion basis and patient basis are given in Table 4 and S2 Table, respectively.

**Table 4:** Intraobserver and interobserver reproducibility in each cohort on a lesion basis in age and gender matched patients.

	Asymptomatic (intra n = 65, inter n = 64)			Acute chest pain–ACS (intra n = 106, inter n = 104)			Acute chest pain + ACS (intra n = 99, inter n = 98)		
	Mean diff±1.96SD	95% CI of mean diff	CV (%)	Mean diff±1.96SD	95% CI of mean diff	CV (%)	Mean diff±1.96SD	95% CI of mean diff	CV (%)
**Intraobserver reproducibility**									
Minimal lumen area, mm^2^	-0.1±2.7	-0.4; 0.3	21	-0.3±2.0	-0.5; -0.1	24	-0.1±2.5	-0.3; 0.2	27
Area stenosis, %	0.2±22.0	-2.6; 3.0	29	0.3±26.7	-2.3; 2.9	32	0.3±18.2	-1.5; 2.2	21
Minimal lumen diameter, mm	0.0±0.5	-0.1; 0.0	11	-0.1±0.5	-0.1; 0.0	14	0.0±0.6	-0.1; 0.0	15
Diameter stenosis, %	0.1±13.7	-1.6; 1.9	31	0.3±20.4	-1.8; 2.3	39	0.2±12.1	-1.1; 1.4	23
Remodeling index	0.0±0.2	0.0; 0.0	11	0.0±0.3	-0.1; 0.0	18	0.0±0.3	0.0; 0.0	19
Eccentricity index	0.0±0.2	0.0; 0.0	10	0.0±0.2	0.0; 0.0	16	0.0±0.3	0.0; 0.0	16
Plaque burden, %	-0.3±7.0	-1.1; 0.6	7	1.3±8.9	0.4; 2.2	8	0.4±10.9	-0.7; 1.5	10
Plaque volume, mm^3^	4.0±52.3	-2.6; 10.6	23	-0.3±47.5	-4.9; 4.4	20	5.5±45.6	0.9; 10.2	20
Dense calcium, mm^3^	0.3±6.3	-0.5; 1.1	13	0.2±6.8	-0.5; 0.8	14	-0.1±6.1	-0.7; 0.5	13
Fibrotic, mm^3^	3.6±28.6	0.0–7.2	24	0.8±29.0	-2.1; 3.6	23	3.4±28.4	0.6; 6.3	23
Fibro-fatty, mm^3^	0.4±14.8	-1.5; 2.3	36	-0.5±14.0	-1.9; 0.8	28	1.4±14.3	-0.1; 2.8	31
Necrotic core, mm^3^	-0.3±12.1	-1.8; 1.2	66	-0.6±6.2	-1.2; 0.0	36	0.8±6.4	0.2; 1.5	42
**Interobserver reproducibility**									
Minimal lumen area, mm^2^	0.4±2.7	0.1; 0.8	22	0.1±2.7	-0.2; 0.3	31	0.3±3.3	-0.1; 0.6	37
Area stenosis, %	2.1±30.6	-1.8; 6.0	41	-1.5±29.4	-4.4; 1.4	35	-0.5±32.3	-3.8; 2.8	37
Minimal lumen diameter, mm	0.1±0.6	0.0, 0.2	12	0.1±0.8	0.0; 0.2	21	0.1±0.9	0.0; 0.2	24
Diameter stenosis, %	1.4±18.7	-1.0; 3.8	43	-0.8±22.3	-3.1; 1.4	44	-0.6±24.7	-3.2; 1.9	46
Remodeling index	0.0±0.3	-0.1; 0.0	14	0.0±0.3	-0.1; 0.0	18	0.0±0.3	-0.1; 0.0	18
Eccentricity index	0.0±0.2	0.0; 0.0	15	0.0±0.3	-0.1; 0.0	16	0.0±0.3	0.0; 0.0	19
Plaque burden, %	-3.7±10.0	-4.9; -2.4	10	-2.5±11.4	-3.6; -1.4	11	-3.8±13.8	-5.2; -2.4	12
Plaque volume, mm^3^	-9.7±66.2	-18.2; -1.3	27	5.6±53.4	0.3; 10.9	23	-2.7±75.2	-10.4; 5.0	31
Dense calcium, mm^3^	-4.1±12.0	-5.7; -2.6	22	-2.9±12.8	-4.2; -1.6	24	-4.4±14.1	-5.9; -3.0	27
Fibrotic, mm^3^	-6.9±41.8	-12.3; -1.6	32	5.3±29.1	2.4;8.2	25	-0.2±43.8	-4.7; 4.3	34
Fibro-fatty, mm^3^	0.3±15.4	-1.6; 2.3	37	2.2±15.9	0.6; 3.8	35	1.4±21.8	-0.9; 3.6	46
Necrotic core, mm^3^	1.0±9.8	-0.3; 2.2	54	0.9±9.4	0.0; 1.9	61	0.5±8.5	-0.4; 1.4	56

ACS acute coronary syndrome; SD standard deviation; CV coefficient of variation; CI confidence interval

Geometrical vessel and lumen parameters: Intraobserver and interobserver reproducibility of MLA and MLD, though acceptable in all subpopulations, decreased with worsening clinical risk profile, as is expressed by widening limits of agreement and increasing CV. Intraobserver reproducibility of area and diameter stenosis was highest in the “Chest pain + ACS” subpopulation. Interobserver reproducibility of area and diameter stenosis, however, did not vary notably across subpopulations. Limits of agreement for RI increased (along with CV) with worsening clinical risk profile.

Plaque eccentricity and plaque burden: Limits of agreement for EI increased (along with CV) with worsening clinical risk profile. Intraobserver and interobserver reproducibility estimates for plaque burden were acceptable in all subpopulations but limits of agreement and CV increased with worsening clinical risk profile.

Plaque volume and plaque composition: Reproducibility of total plaque volume was acceptable in all subpopulations and, for interobserver reproducibility, was highest in the “*Chest pain–ACS”* subpopulation. On a lesion basis, a pattern of worsening reproducibility for plaque composition with decreasing plaque density was seen.

## Discussion

This study, to our knowledge, is the largest comprehensive examination of reproducibility of semi-automated plaque quantification in and across populations with different clinical presentations. The main findings of this study are: Firstly, reproducibility of MLA, MLD, plaque burden, and plaque volumes was acceptable. Reproducibility was poorer for area stenosis, diameter stenosis, RI, and EI assessed on a continuous scale but acceptable using a categorical approach. Secondly, reproducibility varied for compositional measures where coronary plaque with higher densities had better reproducibility than coronary plaque with lower densities. Thirdly, reproducibility of the investigated parameters generally decreased with worsening clinical risk profile. Overall, this study showed that semi-automated plaque quantification provided reproducible assessments of clinically relevant coronary plaque geometry, plaque distribution, plaque burden, and plaque volumes, especially high density plaque volumes.

Our findings on the reproducibility of total plaque volume and low density plaque are comparable with a study by Øvrehus et al[8] who demonstrated an intraobserver and interobserver CV of 8% and 12% respectively for total plaque volume (compared to CV 11% and 17% respectively in the present study). Furthermore, Øvrehus et al documented a CV of 46% (intraobserver reproducibility) and 43% (interobserver reproducibility) for non-calcified plaque volume which is comparable with the findings of reproducibility for fibro-fatty and necrotic core tissue that ranged from 30% to 57% in the present study.

With regards to the poorer reproducibility found in lower plaque densities compared to higher plaque densities, other studies have found similar results[5,9]. Our findings may be due manual editing of the automatic vessel and lumen contours which may have contributed to higher intraobserver and interobserver variability, as has also been reported by Laqmani et al[11] and Blackmon et al[23]. Additionally, as discussed by Papadopoulou et al, decreased reproducibility of non-calcified plaque could be explained by incorrect incorporation of the lumen or pericoronary fat in the plaque area[9].

Across subpopulations, reproducibility of QCT parameters was seen to generally decrease with worsening clinical risk profile. This is an important observation as it relates to the clinical feasibility of QCT in different patient populations. This finding is most likely due to differences in plaque composition and disease burden between the investigated subpopulations. It has been shown that coronary atherosclerotic plaque volume and composition are strongly associated to clinical presentation and several studies have previously reported increased total non-calcified plaque volume and low attenuation plaque volume in patients with ACS compared to patients with stabile angina and asymptomatic individuals[6,7,24].

As discussed, fibro-fatty and necrotic core tissue showed poorer reproducibility compared to fibrous and dense calcium tissue. The greater volumes of lower density plaque in populations with greater likelihood of CAD would, therefore, not only result in poor reproducibility of non-calcified plaque but also of geometrical, distributional, burden and volume related parameters. This is exemplified by the findings of Kang et al who reported a decreased sensitivity to detect diameter stenosis >50% in non-calcified plaque due to underestimation when using automated techniques compared with visual assessments[25]. Furthermore, increased disease burden potentially requires greater manual correction when using semi-automated quantification techniques, thereby introducing greater variability into QCT measurements. This is important with regards to potential implementation of QCT in a clinical setting and emphasizes the importance of accurate automatic identification of especially non-calcified plaque.

At present QCT requires excellent image quality for accurate plaque measurements and, in addition for the need to further automate quantitative coronary plaque assessments, there is also a need for improvements in software technology with regards to the assessment of currently unevaluable coronary segments due to motion, noise, severe calcification, small vessel diameter, stents, etc. Further developments in novel iterative reconstruction algorithms as well as new scanners with higher resolution and rotation speeds, and the use of kV switching and iodine mapping may all improve image quality and increase the feasibility of the automated plaque assessments[26].

### Perspectives

A cornerstone in the assessment of the clinical applicability of QCT is the determination of its reproducibility. This is of particular importance as various studies have found QCT assessments to be of prognostic value[3–5,27]. Furthermore, there is an increased focus on the global assessment of coronary atherosclerotic disease–which can be achieved using QCT—for the identification of the vulnerable patient instead of traditional assessments focusing on the identification potentially vulnerable lesions[28,29]. The findings of this study will, therefore, aid in the further implementation of semi-automated quantitative plaque parameters for risk stratification, especially with regards to global measures of atherosclerotic disease as our findings on patient based coronary plaque volumes were especially good. Limitations regarding plaque composition of fibro-fatty and necrotic core tissue, however, persist. Furthermore, current appropriateness criteria do not recommend contrast-enhanced MDCT in asymptomatic individuals, primarily due to possible adverse effects of x-ray radiation. Our findings regarding the good QCT reproducibility in asymptomatic individuals compared to patients with acute chest pain, however, demonstrate the potential applicability of QCT in asymptomatic individuals, especially since technological advancements of low-dose MDCT now allow for the attainment of a contrast-enhanced MDCT <1 mSv[8].

### Study limitations

Firstly, potential differences in MDCT scan quality between subpopulations cannot be ruled out as the scan protocol for each of the subpopulations varied slightly (due to ethical considerations concerning radiation dose). Systematic differences in image quality between subpopulations would result in differences in reproducibility. Heart rate and BMI was, however, not statistically significantly different between the subpopulations and MDCTs with the lowest radiation doses were of participants in the “*Asymptomatic”* subpopulation–the subpopulation which often had the best reproducibility estimates.

Secondly, lesions in coronary segments affected by motion, noise, severe calcification, small vessel diameter, limited retrograde contrast filling due to occlusion, and stents were not assessed. Improvements in software technology may, in the future, allow for the assessment of such lesions.

Thirdly, as this is a single centre study, generalisability of our findings to other centres, due to possible variations in data acquisition, reading, and software may be reduced.

### Conclusions

This study showed that semi-automated plaque quantification provided reproducible assessments of minimal lumen area, minimal lumen diameter, plaque burden, and plaque volume and acceptably detected area and diameter stenoses ≥50%, positive remodeling, and eccentric plaque. With regards to plaque composition, reproducibility was best for high density plaque volumes. Lastly, across populations with different clinical presentations, reproducibility generally decreased with worsening clinical risk profile.

## Supporting information

S1 Table Intraobserver and interobserver reproducibility estimates on a patient basis. CCC concordance correlation coefficient; SD standard deviation; CV coefficient of variation; CI confidence interval.
(DOCX)

S2 Table Intraobserver and interobserver reproducibility between cohorts on a patient basis in aged and gender matched patients. ACS acute coronary syndrome; SD standard deviation; CV coefficient of variation; CI confidence interval.
(DOCX)
