# Supplementary material for: Reproducibility of quantitative coronary computed tomography angiography in asymptomatic individuals and patients with acute chest pain
Source: PLoS One. 2018 Dec 14;13(12):e0207980. doi: 10.1371/journal.pone.0207980 (PMC6294364; doi:10.1371/journal.pone.0207980)
Supplement: S1 Table — CCC concordance correlation coefficient; SD standard deviation; CV coefficient of variation; CI confidence interval. (DOCX) [file pone.0207980.s001.docx]

S1 Table. Intraobserver and interobserver reproducibility estimates on a patient basis

|  | Intraobserver reproducibility  n=84 | | | | | Interobserver reproducibility  n=84 | | | | |
| --- | --- | --- | --- | --- | --- | --- | --- | --- | --- | --- |
|  | CCC | 95% CI | Mean diff±1.96SD | 95% CI of mean diff | CV (%) | CCC | 95% CI | Mean diff±1.96SD | 95% CI of mean diff | CV (%) |
| Total plaque volume,mm^3^ | 0.99 | 0.989-0.995 | 15.1±100.3 | 4.0; 26.2 | 11 | 0.98 | 0.975-0.989 | 2.8±155.4 | -14.5; 20.0 | 17 |
| Total dense calcium volume, mm^3^ | >0.99 | 0.999-0.999 | 0.9±13.8 | -0.6; 2.5 | 7 | 0.98 | 0.976-0.987 | -12.6±39.2 | -17.0; -8.3 | 20 |
| Total fibrotic volume, mm^3^ | 0.99 | 0.984-0.993 | 11.4±61.1 | 4.6; 18.2 | 13 | 0.98 | 0.962-0.984 | 5.1±95.4 | -5.5; 15.6 | 20 |
| Total fibro-fatty volume, mm^3^ | 0.99 | 0.980-0.992 | 2.3±26.5 | -0.6; 5.3 | 15 | 0.96 | 0.945-0.975 | 6.6±41.9 | 1.9; 11.2 | 24 |
| Total necrotic core volume, mm^3^ | 0.97 | 0.950-0.979 | 0.5±17.0 | -1.4; 2.4 | 26 | 0.94 | 0.906-0.956 | 3.5±21.2 | 1.1; 5.8 | 35 |

*CCC concordance correlation coefficient; SD standard deviation; CV coefficient of variation; CI confidence interval*
