# Supplementary material for: Reproducibility of quantitative coronary computed tomography angiography in asymptomatic individuals and patients with acute chest pain
Source: PLoS One. 2018 Dec 14;13(12):e0207980. doi: 10.1371/journal.pone.0207980 (PMC6294364; doi:10.1371/journal.pone.0207980)
Supplement: S2 Table — ACS acute coronary syndrome; SD standard deviation; CV coefficient of variation; CI confidence interval. (DOCX) [file pone.0207980.s002.docx]

S2 Table. Intraobserver and interobserver reproducibility between cohorts on a patient basis in aged and gender matched patients

|  | **Asymptomatic**  n=21 | | | **Acute chest pain – ACS**  n=21 | | | **Acute chest pain + ACS**  n=21 | | |
| --- | --- | --- | --- | --- | --- | --- | --- | --- | --- |
|  | Mean diff±1.96SD | 95% CI of mean diff | CV (%) | Mean diff±1.96SD | 95% CI of mean diff | CV (%) | Mean diff±1.96SD | 95% CI of mean diff | CV (%) |
| **Intraobserver reproducibility** |  |  |  |  |  |  |  |  |  |
| Total plaque volume, mm^3^ | 12.1±102.7 | -11.8; 35.9 | 15 | 0.2±100.8 | -23.3; 23.4 | 9 | 24.0±80.1 | 5.3; 42.6 | 7 |
| Total dense calcium volume, mm^3^ | 0.9±10.1 | -1.5; 3.2 | 7 | 0.2±15.2 | -3.4; 3.7 | 6 | 0.1±14.5 | -3.2; 3.5 | 7 |
| Total fibrotic volume, mm^3^ | 11.0±56.4 | -2.1; 24.1 | 15 | 4.2±61.2 | -10.1; 18.4 | 11 | 15.1±55.3 | 2.2; 27.9 | 10 |
| Total fibro-fatty volume, mm^3^ | 1.1±28.9 | -5.6; 7.8 | 23 | -1.6±31.1 | -8.8; 5.7 | 14 | 5.4±21.8 | 0.3; 10.4 | 10 |
| Total necrotic core volume, mm^3^ | -1.0±22.3 | -6.2; 4.2 | 40 | -2.4±14.9 | -5.8; 1.1 | 19 | 3.5±9.9 | 1.3; 5.8 | 14 |
| **Interobserver reproducibility** |  |  |  |  |  |  |  |  |  |
| Total plaque volume, mm^3^ | -29.7±116.7 | -56.8; -2.8 | 16 | 29.7±114.4 | 3.1; 56.3 | 10 | -4.6±224.6 | -56.7; 47.6 | 21 |
| Total dense calcium volume, mm^3^ | -12.1±33.3 | -19.9; -4.4 | 33 | -13.8±48.5 | -25.1; -2.6 | 19 | -19.9±42.8 | -29.9; -10.0 | 18 |
| Total fibrotic volume, mm^3^ | -21.3±91.8 | -42.6; 0.0 | 24 | 27.3±71.0 | 10.8; 43.8 | 13 | 4.5±119.0 | -23.2; 32.1 | 22 |
| Total fibro-fatty volume, mm^3^ | 0.8±23.8 | -4.7; 6.4 | 19 | 11.0±33.8 | 3.2; 18.9 | 16 | 8.1±65.0 | -7.0; 23.2 | 32 |
| Total necrotic core volume, mm^3^ | 2.9±18.4 | -1.4; 7.2 | 34 | 4.7±24.8 | -1.1; 10.4 | 33 | 2.5±22.5 | -2.7; 7.7 | 35 |

*ACS acute coronary syndrome; SD standard deviation; CV coefficient of variation; CI confidence interval*
